# Tensile Properties and Mechanism of Carbon Fiber Triaxial Woven Fabric Composites

**DOI:** 10.3390/ma18133154

**Published:** 2025-07-03

**Authors:** Yunfei Rao, Chen Zhang, Miao Yi

**Affiliations:** 1 College of Textile Science and Engineering, International Institute of Silk, Zhejiang Sci-Tech University, Hangzhou 310018, China; 2 College of Textiles, Donghua University, Shanghai 201620, China; 3 Shanghai Science and Technology Exchange Center (SSTEC), Shanghai 200030, China

**Keywords:** triaxial woven fabric, carbon fiber, composite molding, tensile property, interweaving points, damage mode, yarn spreading

## Abstract

The manufacturing methodologies for carbon fiber triaxial woven fabric composites demonstrate significant variability, resulting in the failure mechanisms under tensile loading conditions, and the fundamental role of interweaving points remains unclear. Moreover, the mechanisms of destruction under tensile loads have not been sufficiently studied. In this study, the resin transfer molding and resin film infusion were selected to fabricate carbon fiber triaxial woven fabric composites, with a specific focus on their effects on the tensile properties of carbon fiber triaxial woven composites. Compared with ordinary materials, the tensile load of carbon fiber triaxial woven fabric composites after yarn spreading has increased by more than 30%. The strength can reach 1133 MPa after yarn spreading of 3k carbon fiber, which was 39% higher than the original. Furthermore, acoustic emission monitoring shows that the counts of acoustic signals in the first half dropped from 10,000 to around 3000, mostly due to the reduction of resin and fiber/matrix debonding. The digital image correlation provided full-field strain analysis, which proved that the strain of the fibers at the interweaving points decreased significantly during the stretching process after yarn spreading.

## 1. Introduction

Triaxial woven fabric, which consists of two sets of warp yarns and one set of weft yarns interlaced at 60° angles, forms a stable hexagonal pore structure [1]. This unique architecture results in nearly isotropic in-plane mechanical properties. Compared to conventional biaxial woven fabrics (comprising only warp and weft yarns), triaxial woven fabrics demonstrate superior load transfer, effectively mitigating stress concentration while resisting deformation [2,3,4,5]. These characteristics significantly enhance the fatigue resistance and impact performance of materials [6,7]. The traditional woven fabric and the triaxial woven fabric prepared with the same material, although the weight per square meter of the latter has decreased by 33%, its tear strength has increased by more than 70% [8].

Triaxial woven fabric composites demonstrate exceptional mechanical properties due to their unique architecture and the intrinsic advantages of high-performance reinforcing fibers such as carbon and aramid. These characteristics, including superior specific strength and stiffness, make them ideally suited for applications demanding optimal strength-to-weight ratios [9]. As a result, these advanced composites have gained significant traction in aerospace and defense applications where weight reduction and structural performance are paramount [10,11]. Beyond their standalone applications, triaxial woven fabrics serve as excellent reinforcement materials for hybrid composite systems. Their distinctive hexagonal open-cell structure contributes to weight reduction while maintaining mechanical performance comparable to denser fabric architectures [12,13]. The single-layer weight of the carbon fiber triaxial woven fabric (0.025 g/cm^2^) reinforced unidirectional fabric (0.075 g/cm^2^) laminate composite was reduced by two-thirds, and the bending performance can be maintained at more than 99% [14].

The architectural configuration of triaxial woven fabrics can be precisely engineered to satisfy specific performance requirements. In our previous research, we developed dedicated manufacturing equipment specifically designed for carbon fiber triaxial woven fabrics. Through systematic investigation, we established a comprehensive structural model that correlates key weaving parameters with resultant fabric geometries. This model demonstrates that by strategically adjusting weaving parameters, we can controllably produce fabrics with varying hexagonal aperture dimensions and distinct interlocking cross-sectional architectures. These fundamental structure–property relationships serve as valuable guidelines for the targeted fabrication of carbon fiber triaxial woven fabrics optimized for diverse engineering applications [15].

Vacuum-assisted resin infusion molding coupled with thermal curing currently stands as the predominant manufacturing technique for carbon fiber reinforced composites [16,17]. However, this process presents significant challenges when applied to triaxial woven fabrics, as the hexagonal pore structures frequently become entirely resin-saturated. This phenomenon leads to suboptimal fiber volume fractions and promotes resin-rich zones that become preferential failure sites under tensile loading conditions [18,19]. While carbon fibers predominantly govern the composite strength, premature resin matrix failure significantly compromises the structural integrity of the material system [20,21].

The current research landscape on triaxial woven fabric composites has largely concentrated on macroscopic mechanical properties and failure patterns [22,23], with limited fundamental understanding of the underlying damage mechanisms. Critical knowledge gaps persist regarding the distinct failure contributions from resin matrix versus carbon fiber reinforcement and the progressive damage evolution at fiber–resin interfaces [24,25]. Although prior studies have recognized the mechanical significance of fiber interlocking points in triaxial architectures, the dynamic evolution of these critical structural features during tensile loading and their precise role in damage progression remain poorly characterized.

Herein, we developed an enhanced manufacturing methodology integrating resin film infusion with vacuum-assisted processing, successfully fabricating carbon fiber triaxial woven fabric composites with lower void content. A systematic comparative analysis was performed between composites manufactured using this optimized approach and those produced via conventional methods. Compared with ordinary materials, the tensile load of carbon fiber triaxial woven fabric composites after yarn spreading has increased by more than 30%. The strength can reach 1133 MPa after yarn spreading of 3k carbon fiber, which was 39% higher than the original. Moreover, acoustic emission monitoring shows that the counts of acoustic signals in the first half dropped from 10,000 to around 3000, mostly due to the reduction of resin and fiber/matrix debonding. The digital image correlation provided full-field strain analysis, which proved that the strain of the fibers at the interweaving points decreased significantly during the stretching process after yarn spreading.

## 2. Experimental Section

Materials: The carbon fibers used are 3k, 6k, and 12k of T700 from TORAY Inc. (Tokyo, Japan), and the resin film is ZW-01 from JFTX Inc. (Shanghai, China) in this work. The resin used for observing the cross-section of materials under a metallographic microscope is 2511-A/BS.

Preparation: The carbon fiber yarn spreading process was conducted on the JFTX Inc. production line using a fiber expansion mechanism comprising alternating upper and lower fiber expansion rollers and an infrared heating device, where adjustable roller positions regulated both the enclosure angle between the carbon fiber and rollers and the fiber tension. Following yarn spreading, the fibers underwent stretching and curling before being woven into triaxial carbon fiber fabrics using laboratory-made looms equipped with a warp feeding mechanism, with the detailed weaving process referenced from prior research. Specific details can be found in Supporting Information.

Characterization: The tensile test refers to the standard ASTM-D3039 [26], and the width of the specimen is a single cell of the triaxial structure. The tensile testing of composites for different specifications was accomplished by using the universal testing machine (Lanbo-Sansi Inc., Shenzhen, China). Prepare five samples of each type for testing and take the average value. Acoustic Emission (Physical Acoustics Corporation, Princeton Junction, NJ, USA) and Digital Image Correlation methods (Correlated Solutions Inc., Irmo, SC, USA) were used to analyze the failure modes of composites and the strain of composites, respectively. Specific details can be found in Supporting Information.

## 3. Results and Discussion

**Figure 1:**
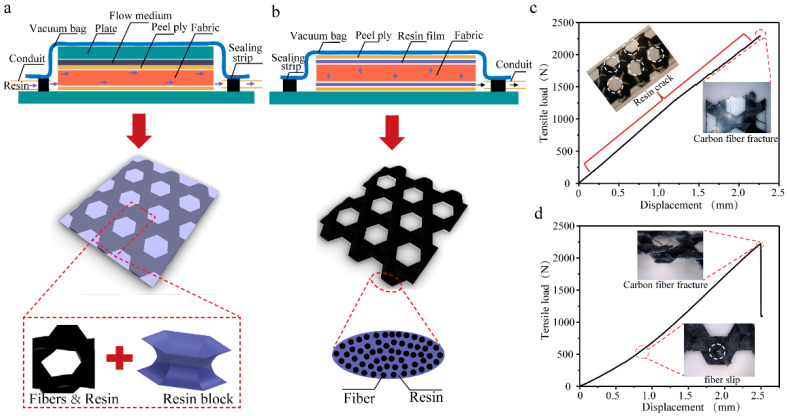
The forming of carbon fiber triaxial woven fabric composites. (**a**) Resin transfer molding. (**b**) Resin film infusion molding. (**c**) The tensile load–displacement of resin transfer molding. (**d**) The tensile load–displacement of resin film infusion molding. Note that the blue and black arrows in a and b represent directions of resin and air, respectively. The white circles in c and d represent damages of composites.

Composites Forming. Two distinct manufacturing approaches (resin transfer molding and resin film infusion) were employed to fabricate carbon fiber triaxial woven fabric composites. The fundamental difference between these methods lies in their resin delivery mechanisms. In the resin transfer molding process, liquid resin was injected into the dry fabric stack under vacuum conditions, with two mold plates serving as the primary flow channels (Figure 1a). This approach resulted in complete fiber impregnation but led to the formation of resin-rich zones within the hexagonal pore structures due to unrestricted resin flow. In contrast, the resin film infusion process utilized pre-positioned resin films that melted during heating to form a viscous resin phase. The molten resin gradually infiltrated the carbon fiber reinforcement through capillary action and applied pressure. During curing, progressively increasing air pressure combined with vacuum bag consolidation ensured intimate contact between the resin and fibers (Figure 1b). This controlled infusion process effectively eliminated the hexagonal pore structure characteristic of the triaxial fabric architecture. Additional process details and characterization data are provided in the Supporting Information.

The tensile strength comparison between composites fabricated using the two forming methods revealed no significant difference (Figure 1c), demonstrating that carbon fibers predominantly govern the tensile behavior. However, distinct failure mechanisms were observed while composites produced via resin film infusion exhibited noticeable fiber slippage at interweaving points during tensile loading, resulting in slightly increased displacement (Figure 1d). In contrast, resin transfer molded composites demonstrated constrained fiber movement due to complete resin encapsulation, yielding straighter stress–strain curves. This resin-dominated matrix system experienced progressive fracture during tensile deformation, ultimately compromising material integrity. Based on these findings, the resin transfer method was selected as the optimal approach for curing both standard and spread-out carbon fiber triaxial woven fabrics. For clarity, specimen nomenclature is defined as follows: T-3k, T-6k, and T-12k denote triaxial woven fabric composites utilizing 3K, 6K, and 12K carbon fibers, respectively, while S-3k, S-6k, and S-12k represent their yarn spread counterparts.

### 3.1. Tensile Properties

**Figure 2:**
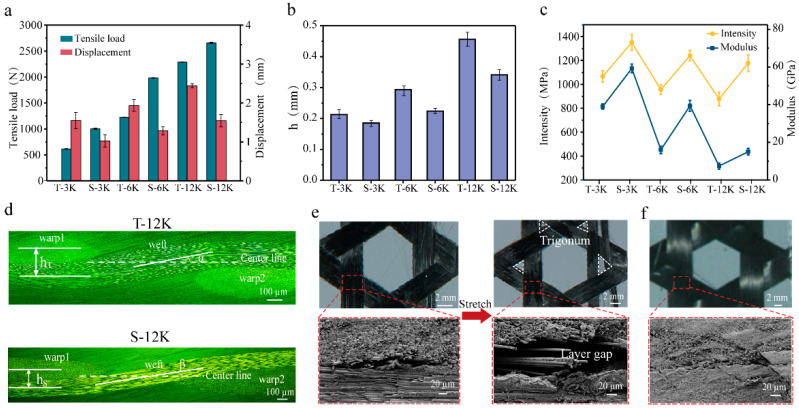
Tensile properties of carbon fiber triaxial woven fabric composites. (**a**) Tensile load–displacement, flexural wave height (**b**), and intensity modulus (**c**) of different composites. (**d**) Cross-section of T-12k and S-12k. (**e**) Fiber sliding and delamination of T-12k. (**f**) S-12k in stretching.

Figure 2a demonstrates the tensile load–displacement behavior of triaxial carbon fiber woven composites with varying fiber specifications, revealing that both maximum tensile load and displacement capacity exhibit positive correlations with increasing carbon fiber “k” numbers. This mechanical enhancement primarily originates from the greater number of load-bearing fibers within wider yarns that collectively resist tensile forces. Simultaneously, the elevated fiber count induces larger tensile strain, predominantly attributed to amplified buckling deformation at interweaving points. As illustrated in Figure 2b, yarns with higher fiber counts develop thicker cross-sections that undergo more pronounced buckling at interweaving regions—a phenomenon that corroborates our established geometric model. This buckling mechanism enables greater displacement accommodation during tensile loading, particularly at critical interweaving points. However, the accompanying fiber slippage during deformation creates a non-uniform stress distribution among individual fibers, ultimately leading to the observed substantial reduction in tensile strength and moderate modulus degradation with increasing yarn width, as quantitatively shown in Figure 2c.

The flexural wave height, defined as the vertical distance between adjacent warp yarn centers in the cross-section of carbon fiber triaxial woven fabric, serves as a critical geometric parameter influencing mechanical performance. To systematically investigate the relationship between buckling degree and mechanical properties, this study employs spread tow triaxial carbon fiber woven composites (S-12k) as a comparative system. Experimental results demonstrate that the spread tow configuration yields a substantial 35% improvement in maximum tensile load compared to conventional carbon fiber yarn composites (T-12k), while exhibiting a corresponding 20% reduction in displacement capacity. This mechanical behavior directly correlates with the increased number of effectively load-bearing fibers achieved through the spreading process, which enhances load transfer efficiency during tensile deformation while constraining yarn mobility at interweaving points.

Notably, the enhancement effect exhibits a distinct inverse correlation with fiber k-number, with spread tow 3k, 6k, and 12k composites demonstrating progressively diminishing tensile load improvements of 63.4%, 45.8%, and 33.8%, respectively (Figure 2c). This phenomenon likely stems from geometric constraints in the yarn spreading process, as the initial fiber count decreases, the potential for achieving optimal yarn flatness becomes increasingly constrained, thereby reducing interweaving point buckling and enabling more efficient load transfer under applied stresses. In contrast to the k-number-dependent load enhancement, the displacement reduction remains consistently around 30% across all fiber specifications, a stability that is similarly mirrored in the material’s strength and modulus improvements, suggesting that while the spreading process’s effectiveness diminishes with increasing fiber count, its fundamental mechanical influence remains consistent regardless of yarn size.

As illustrated in Figure 2d, the cross-sectional analysis reveals a fundamental structural distinction between conventional (T-12k) and spread tow (S-12k) triaxial woven composites: the latter exhibits significantly reduced interweaving degree between warp and weft yarns along with a markedly smaller vertical spacing between adjacent warp yarns (h_S_ < h_T_). This optimized architecture facilitates superior fiber alignment through enhanced inter-yarn slippage during loading, thereby mobilizing a greater proportion of fibers for vertical load-bearing. Importantly, while conventional wider carbon fiber yarns typically suffer from compromised tensile strength and modest modulus reduction, the spreading treatment successfully counteracts these detrimental effects. The deformation characteristics are further elucidated in Figure 2e,f, where comparative analysis of T-12k specimens demonstrates progressive formation of extensive triangular zones at interweaving points during stretching (from initial to 80% maximum strain), resulting from pronounced fiber sliding and inter-yarn delamination. In striking contrast, spread tow composites maintain structural integrity even at 50% maximum strain, with no observable triangular slip zones or delamination, unequivocally demonstrating that significant material damage and failure are predominantly delayed to the final stages of tensile deformation.

### 3.2. Damage Modes

**Figure 3:**
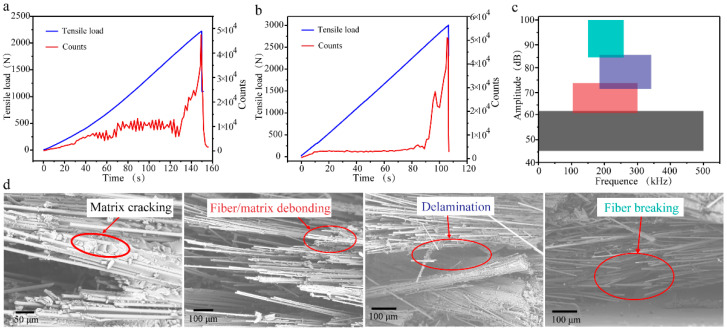
Acoustic emission signals and failure modes. (**a**) AE counts of T-12k. (**b**) AE counts of S-12k. Four types of acoustic emission signals (**c**) and corresponding damages of composites (**d**).

To investigate the fracture behavior of carbon fiber triaxial woven fabric composites under tensile loading, acoustic emission (AE) monitoring was conducted on both conventional 12k carbon fiber (T-12k) and spread yarn (S-12k) composites. The AE response profiles revealed distinct damage progression characteristics between the two material systems. As depicted in Figure 3a, T-12k specimens exhibited gradual AE count accumulation during initial loading, followed by pronounced fluctuations between 4000~12,000 counts in the intermediate phase, culminating in rapid signal escalation and subsequent decay during final failure. In contrast, S-12k composites (Figure 3b) maintained relatively stable AE activity throughout early and mid-stage loading, with only minor fluctuations preceding the abrupt signal surge at ultimate failure. While both materials demonstrated comparable maximum AE counts and similar rapid signal collapse post-failure, T-12k displayed more substantial count variations during initial deformation. This AE behavior correlates with the observed mechanical response, where spread yarn composites exhibited more consistent tensile curve slopes, whereas T-12k showed progressively increasing load growth rates throughout the test.

Through systematic characterization of tensile-induced failure modes, we identified four distinct damage mechanisms: resin cracking, fiber–matrix debonding, delamination, and fiber fracture. Building upon experimental AE signatures and validated literature correlations [27,28], we developed a robust classification scheme based on amplitude and peak frequency characteristics (Figure 3c). Our analysis reveals that resin cracking generates the lowest-amplitude AE signals (<60 dB), while fiber–matrix debonding produces moderate-amplitude (60~75 dB), low-frequency (50~150 kHz) signals. Delamination events are characterized by higher-amplitude (75~90 dB) and higher-frequency (150~300 kHz) signatures, with fiber fracture yielding the most intense signals (>90 dB). This quantitative classification enables precise, real-time monitoring of failure progression throughout tensile loading (Figure 3d), establishing a reliable framework for in situ damage characterization in composite structures. The demonstrated correlation between specific AE parameters and underlying failure mechanisms provides a powerful diagnostic tool for structural health monitoring applications.

**Figure 4:**
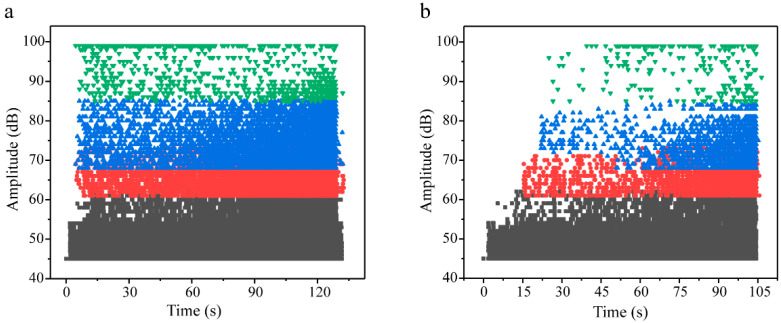
Damage modes of T-12k (**a**) and S-12k (**b**) during stretching process. Note that the black, red, blue and green represent matrix, fiber/matrix debonding, delamination and fiber breaking, respectively.

As evidenced in Figure 4, both conventional carbon fiber triaxial woven composites (T-12k) and their spread yarn counterparts (S-12k) exhibited continuous resin fracture events throughout the entire tensile loading process until final material failure, where black, red, blue, and green markers, respectively, denote matrix cracking, fiber/matrix debonding, delamination, and fiber fracture signals. The key distinction lies in the significantly higher frequency of fiber–matrix debonding and yarn delamination events in conventional T-12k composites, along with the persistent occurrence of fiber fracture signals from initial to final loading stages despite their relatively low overall proportion. In contrast, spread yarn composites (S-12k) demonstrated delayed onset and substantially reduced occurrence of these critical failure modes—fiber–matrix debonding, yarn delamination, and fiber fracture signals predominantly emerged immediately preceding ultimate failure. This temporal shift in damage progression reveals that the extensive fiber–matrix debonding and delamination in T-12k composites actively promoted premature fiber fracture, whereas the optimized architecture of S-12k composites effectively postponed fiber failure until the advanced loading stages, highlighting the superior damage tolerance achieved through yarn spreading modification.

### 3.3. Stretching Mechanism

**Figure 5:**
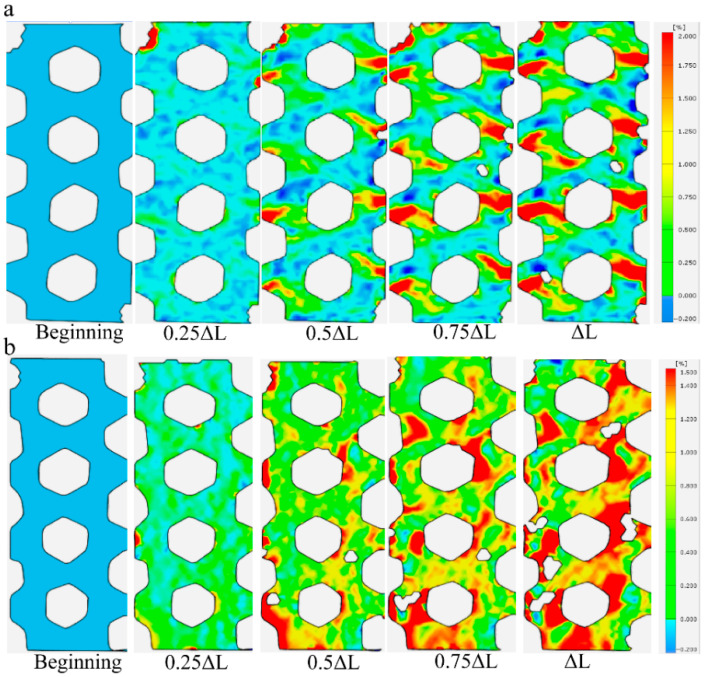
The strain of composite. (**a**) T-12k. (**b**) S-12k.

Given that the failure mode of carbon fiber triaxial woven fabrics changed after yarn spreading, and the slippage and delamination failure between fibers mainly occurred at the interweaving points, we can use optical measurement methods to monitor the strain at various locations of the fibers in real time during the stretching process. At the beginning stage of stretching for original carbon fiber triaxial woven fabrics, the strain of the specimens tended to be in a stable state, and the degree of strain that occurred was relatively small. With the continuous increase in the load, the strain at different positions on the material surface gradually varies. The interweaving points of the triaxial woven fabrics were the areas where the material strain was the greatest (Figure 5a). However, the strain in other areas outside the interweaving points was also obvious in the carbon fiber triaxial woven fabrics after yarn spreading (Figure 5b). That was because the fibers in the material were straightened more after yarn spreading and weaving, with smaller buckling at the interweaving points. During the stretching process, the fibers can better resist external forces in coordination, and the slip generated at the interweaving points is smaller.

**Figure 6:**
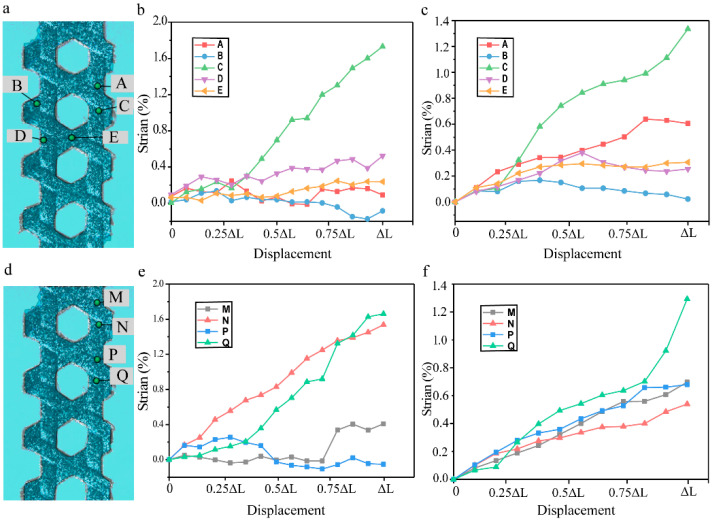
The strain at different points on the composites. (**a**) The selection points around the hexagonal holes of T-12k (**b**) and S-12k (**c**). (**d**) The selection points in vertical direction of T-12k (**e**) and S-12k (**f**).

Through comprehensive strain field analysis at strategically selected material points, we observed distinct strain distribution patterns during tensile loading. As shown in Figure 6a, Points A and C, representing adjacent interweaving points in conventional carbon fiber triaxial woven fabric composites, exhibited markedly different strain responses—point C demonstrated pronounced strain evolution while point A remained relatively stable, revealing a competitive relationship between neighboring interweaving points where proximity to the fixture determines strain priority. Systematic measurements confirmed that all interweaving points undergo intermittent strain concentration, with the magnitude of this effect directly correlating with the degree of buckling deformation. Notably, conventional composites displayed significant yarn slippage absent in their spread yarn counterparts, leading to pronounced Poisson-induced lateral contraction that manifested as negative strain at specimen edges (point B) during advanced loading stages. Comparative analysis of points D and E on the same horizontal plane further revealed load direction dependence, where point D (aligned with the loading axis) experienced greater strain accumulation than point E (located on transverse warp yarns) due to differential load transfer efficiency, with measured strain differences reaching 15~20% at peak load. These findings collectively demonstrate how localized architectural features govern global strain distribution in triaxial woven composites.

To further investigate the strain distribution characteristics around interweaving points, we conducted a comparative analysis of vertically aligned measurement points positioned at the upper and lower extremities of these critical regions in both material types (Figure 6b). The conventional triaxial woven composite (T-12k) exhibited pronounced strain disparities between paired measurement points (M vs. N and P vs. Q), with differentials exceeding 25% at peak load. In stark contrast, the yarn spread composite (S-12k) demonstrated markedly reduced strain variation between corresponding point pairs. This fundamental difference stems from the distinct structural architectures—the reduced thickness and minimized yarn buckling in spread tow fabrics create more homogeneous stress distribution during loading. The S-12k’s flatter, more uniform yarn configuration promotes cooperative load sharing among all three yarn systems, effectively eliminating localized strain concentrations that characterize conventional triaxial weaves. These findings conclusively demonstrate that yarn spreading transforms the deformation mechanism from discrete, point-specific strain accumulation to integrated, system-wide stress redistribution, thereby fully realizing the synergistic potential of the triaxial fiber network.

## 4. Conclusions

In this study, we found that employing the resin film infusion molding process to fabricate triaxial woven fabric composite effectively prevents resin accumulation in hexagonal voids, thereby mitigating material failure caused by resin cracking during tensile loading. Comparative analysis with spread tow triaxial woven fabric composite, combined with acoustic emission monitoring, demonstrates that the tensile properties have been increased, while the intensity can reach 1133 MPa after yarn spreading of 3k carbon fiber, which was 39% higher than the original. Because of the reduction of resin and fiber/matrix debonding in the first half of stretching, the counts of the acoustic signal dropped from 10,000 to around 3000. Furthermore, strain field analysis indicates that reduced buckling deformation enhances fiber synergy in load-bearing, leading to more uniform stress distribution. This effect substantially alleviates localized strain concentration phenomena in the composite structure.

## Data Availability

The original contributions presented in this study are included in the article/Supplementary Material. Further inquiries can be directed to the corresponding author.

## Supplementary Materials

The following supporting information can be downloaded at: https://www.mdpi.com/article/10.3390/ma18133154/s1, Figure S1. Yarn spreading process of carbon fiber; Figure S2. Weaving equipment for triaxial woven fabric of carbon fiber; Figure S3. Testing for carbon fiber of triaxial woven fabric composites; Table S1. Parameters setting of AE signal acquisition system. Reference [14] is cited in supplementary materials.
