# Supplementary material for: Tensile Properties and Mechanism of Carbon Fiber Triaxial Woven Fabric Composites
Source: Materials (Basel). 2025 Jul 3;18(13):3154. doi: 10.3390/ma18133154 (PMC12250782; doi:10.3390/ma18133154)
Supplement: Supplementary file 1 [file materials-18-03154-s001.zip › materials-3715069-supplementary.pdf]

Supplementary material to:

Tensile Properties and Mechanism of Carbon Fiber Triaxial Woven Fabric Composites

Rao et al.

Spreading process

The carbon fiber yarn spreading process was completed on the production line of J JFTX Inc, as shown in the Fig S3. The specific process was that the carbon fiber yarn was first unwound and then entered the fiber expansion area. The fiber expansion mechanism consisted of two parts: the fiber expansion rollers arranged alternately up and down and the infrared heating device. The fiber spreading rollers can adjust their respective up and down positions, thereby regulating the enclosure angle between the carbon fiber and the fiber spreading rollers and the fiber tension. After the yarn spreading was completed, stretching and curling were carried out.

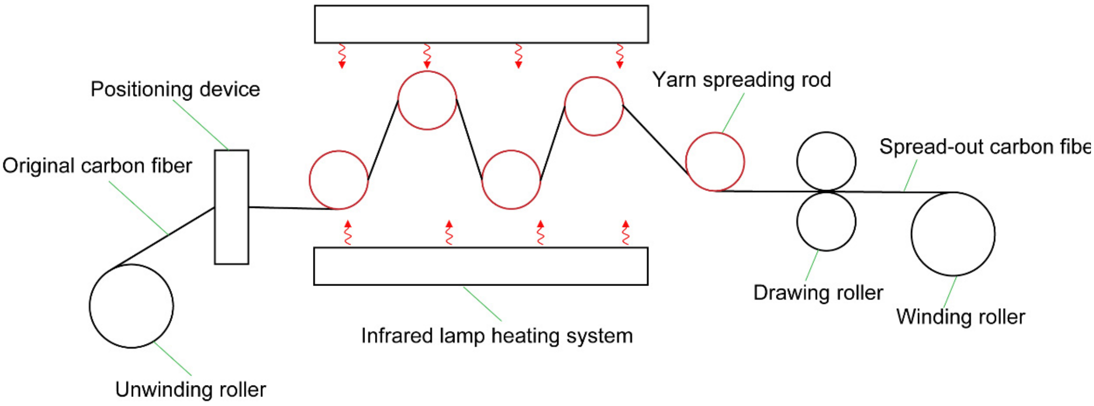

Figure S1. Yarn spreading process of carbon fiber

Weaving process

Carbon fiber triaxial woven fabrics and their corresponding spread-out fabrics were woven by using laboratory-made carbon fiber triaxial woven looms, which include a warp feeding mechanism. The specific weaving process can be referred to our previous research [1].

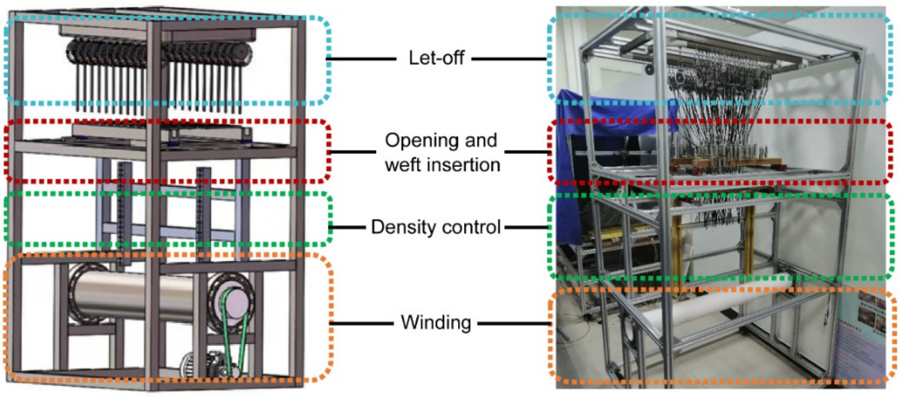

Figure S2. Weaving equipment for triaxial woven fabric of carbon fiber.

Composite forming

The encapsulation layer was peeled off from the resin films. The resin films, still attached to their release papers, were then applied to both upper and lower surfaces of the TWF of carbon fiber. The fabric-resin film assembly was properly laid up in the mold, followed by placement of a breather cloth on top. The entire setup was sealed using a vacuum bag and sealing tape. Following successful vacuum sealing, the system was allowed to stand for 30 minutes to verify the absence of any leaks. The encapsulated mold assembly was then transferred to an oven set at 80°C for thermal curing. Under maintained vacuum conditions, the resin films gradually melted upon heating, transforming into a flowable resin phase. As the mold temperature increased, the molten resin thoroughly impregnated the fabric reinforcement. Excess resin was effectively absorbed by the breather cloth, ensuring complete resin penetration without pore formation in the final composite structure.

Characterization

The tensile, acoustic emission and DIC tests of the specimen were carried out simultaneously, as shown in the Fig S3. The width of the tensile specimen was one unit cell of the triaxial woven fabric, and the length and test steps were in accordance with the American standard ASTM-D3039. The DIC test required the preparation of spots on the specimen using commercial spray glue, and it was necessary to ensure that more than two pictures could be collected per second. In addition, acoustic emission collectors were adhered to the upper and lower sides of the sample. The parameter Settings of the signal collectors are shown in Table S1.

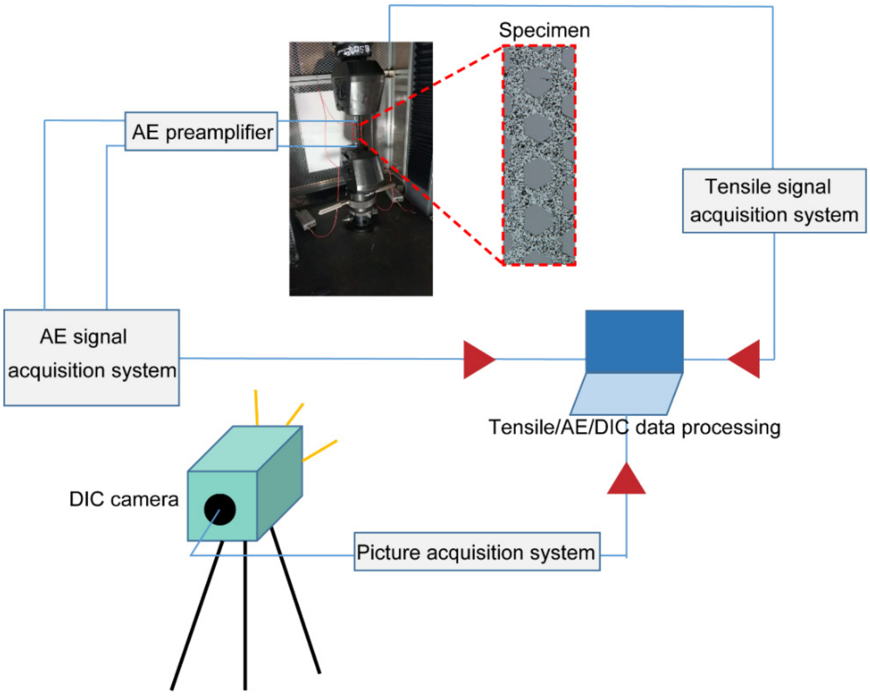

Figure S3. Testing for carbon fiber of triaxial woven fabric composites.

Table S1. Parameters setting of AE signal acquisition system

Parameters	value
Pre-amp gain	40 dB
Sampling frequency	1MSPS
Signal length	2 KB
Threshold	45 dB

References

[1] RAO Y, LI Z, ZHU F, et al. Warp control devices and tension for triaxial woven fabric with carbon fiber [J]. Textile Research Journal, 2023, 93(4482 - 91.
